# Durability of Cement and Ash Mortars with Fluidized and Siliceous Fly Ashes Exposed to HCl Acid Environment over a Period of 2 Years

**DOI:** 10.3390/ma14123229

**Published:** 2021-06-11

**Authors:** Elżbieta Janowska-Renkas, Agnieszka Kaliciak, Grzegorz Janus, Jolanta Kowalska

**Affiliations:** Faculty of Civil Engineering and Architecture, Opole University of Technology, Katowicka Str. 48, 45-061 Opole, Poland; agnieszka.kaliciak10@gmail.com (A.K.); grzegorz_janus@wp.pl (G.J.); j.kowalska@po.opole.pl (J.K.)

**Keywords:** fly ash from fluidized bed combustion (FBC), siliceous fly ash (FA), cement–ash mortars, pozzolanic activity, hydration heat, compressive strength, linear and mass changes, porosity

## Abstract

The paper presents results of research on the impact of fly ash from fluidized bed combustion (FBC) of lignite, used in quantities of 30 and 45% by mass, and the mixture of FBC and silicious fly ash in amount of 45% by mass, on properties of cement–ash mortars. Mortars were exposed to aggressive environment of 1, 3, and 5% HCl solutions for 2 years. Mortars containing 45% FBC exposed to 1% HCl solution (pH = 2) showed the highest durability from among other mortars. The growth of their strength observed after 90 days of testing in 1% HCl environment, as well as the lowest drop of strength after 730 days of exposure to this environment, resulted from the reduced amount of large pores from 20 to 200 nm in mortars containing fly ash, with simultaneous growth of smaller pores of <20 nm during testing. A beneficial effect has been demonstrated of FBC addition to cement on properties of cement–ash mortars exposed to the aggressive impact of the HCl. Mortars with FBC fly-ash content increased to 45% by mass showed higher strength values, smaller differences in linear and mass changes, and increased durability in an aggressive environment observed during 730 days of testing.

## 1. Introduction

Durability of materials based on cement binders may be at risk in the presence of an aggressive environment of chloride ions. This was shown by the results of tests conducted by Rovnanikova [1], based on which the authors demonstrated that durability of cement-based composites was more endangered in the presence of chloride ions originating from MgCl_2_ and NaCl salts than in the presence of Cl^-^ ions originating from hydrochloric acid (HCl).

In the natural environment, the chloride corrosion of cementitious materials may be caused by the impact of humic acids, acid rains, and seawater [2], as well as activity of industry associated with generation of municipal wastewater [2]. Reinforced concrete is mainly used for such structures; hence, when considering the effects of chloride aggression on concrete, one cannot forget the reinforcement steel also exposed to the negative impact of chlorides [3,4]. In order to determine the lifespan of structures exposed to aggressive environments, research is being carried out to develop methods that predict when concrete elements will be damaged and the time of their repair or replacement [5].

A delay in corrosion progress was observed by the authors of [6], who analyzed concrete beams with steel reinforcement. Nevertheless, the applied reinforcement steel also required protection against chloride corrosion [7]. Therefore, the resistance of concrete to the aggressive impact of chlorides can also be increased by using a properly modified composition of the concrete mix (application of mineral additives and chemical admixtures to reduce porosity, and thus water absorption) [8,9].

In consideration of the above, designing of concretes with increased resistance to chloride ion aggression is reasonable. Such concretes, which are particularly exposed to a destructive impact of chlorides, may become a good solution in the future as the structural material used in, e.g., coastal structures, wastewater tanks, or construction of road surfaces. Concrete resistance to chloride ion aggression has been a subject of investigation of many authors [10,11,12].

It is possible to obtain cement composites with enhanced durability and a low content of capillary pores, especially within the paste/aggregate contact zone, where the higher content of crystalline calcium oxide (Ca(OH)_2_) occurs, through reduction of permeability and thus an increase in their resistance to aggressive chloride ions. Chloride aggression, in particular acid aggression, is dangerous for durability of the C-S-H gel [13,14,15,16]. Chlorides of acid origin react with clinker phases, forming easily leachable salts; e.g., CaCl_2_ [16]. Cementitious mortars and concretes with addition of fly ash are high alkaline materials with a pH value amounting to 12–13 that easily react with acidic substances. Solutions with pH < 6 are an aggressive environment for concretes and mortars, leading to their corrosion by pH value reduction below 11 and upsetting the balance within a Ca(OH)_2_ buffer zone [17,18].

In order to increase durability of concrete structure elements exposed to a corrosive impact of chloride ions, it is possible to apply cements with higher SO_3_ content [19]. However, the excessive amount of sulfur oxides (above 3.5%) may lead to delayed expansion in the concrete [20], which in consequence reduces the material’s durability [21]. Calcium chloride, formed in the reaction of chloride ions with calcium hydroxide (1), reacts with constituents of the cement matrix in line with the following reaction Formula (1) [10]:

Ca(OH)_2_ + CaCl_2_ + H_2_O → Ca(OH)_2_CaCl_2_·H_2_O, (1)

As a result of the reaction of hydrated calcium aluminates with calcium chloride, Friedel’s salt is crystallized, and secondary calcium hydroxide is formed. Friedel’s salt may also crystallize in the reaction of a monosulfate with calcium chloride. That reaction is also accompanied by ettringite formation without a change in volume of hydrates. Formation of gypsum and a change in volume accompany the reaction of the monosulfate with calcium chloride [10], whereas in concentrated solutions of chlorides, not gypsum dihydrate, but anhydrite is crystallized [22]. Sulfate ions from chloride ion reactions with monosulfate may react with calcium chloride, including ettringite formation (2) [10]:

4(C_3_A·CaSO_4_·12H_2_O) + 3CaCl_2_ → C_3_A·3CaSO_4_·32H_2_O + 3(C_3_A·CaCl_2_·10H_2_O) + CaSO_4_, (2)

Bernardo et al. [23] showed that formation of ettringite crystals in cement–ash pastes based on FBC fly ash (from fluidized bed combustion) was progressing in an environment with a neutral pH reaction (pH = 7). FBC fly ash contains significant amounts of anhydrite (CaSO_4_) and aluminosilicates [24]. Crystallization of secondary ettringite, caused by addition of FBC fly ash to cement, may lead to linear changes of hardened material, and in consequence to its destruction [25,26,27]. Uncontrolled expansion of hardened cement paste deteriorates tightness of mortars and concrete, which in turn facilitates migration of aggressive ions deep inside the cement matrix, accelerating destruction of the cement composite [14,18,28,29].

The literature also presents results of research that indicate that the phenomenon described above does not occur, and application of materials containing anhydrite is harmless in terms of stability of cement volume with FBC fly ash [30]. Gazdiča et al. [31] claimed, however, that proper grinding of FBC fly ash (including bottom FBC ash) leads to crystallization of ettringite, which does not cause dangerous changes in volume, and portends well for possible utilization of combustion byproducts, such as FBC fly ash in cement-based materials, in the near future.

The use of fly ash from fluidized bed combustion of coal as an additive to cement is not currently allowed by European standards, mainly because of the increased quantity of sulfur oxides in their composition. However, the views on this matter are divided all the time. Some authors, such as Conn et al. [32], showed a lack of possibility for FBC fly-ash application as an additive to cement due to a high content of sulfates in their composition, while Chugh et al. [33], Glinicki [34], and Zieliński [35] presented results of tests indicating that application of FBC ash in cement was possible in proper ratios. Combustion of coal in fluidized bed boilers has a significant impact on the change of fly ash physical and chemical properties. Papers [13,30,36] showed that combustion of coal at a lower temperature (ca. 850 °C) than in pulverized fuel fired boilers (ca. 1200–1400 °C) led to a significant change in FBC fly-ash phase composition. FBC fly ashes showed a higher content of crystalline phases compared to the glassy structure of CFA. Grains of FBC fly ash have an irregular shape compared to grains of ash obtained from pulverized coal fired boilers, which have a spherical shape. Compared to siliceous fly ash (CFA), the structure of fluidized fly ash (FBC) is more amorphous, and its particles have a large specific surface area (approximately 8000 cm^2^/g according to Blaine) [19].

The impact of siliceous fly ash on porosity of cement pastes was widely discussed in [37,38,39]. Singh et al. showed that the use of silica fly ash reduced porosity of cement–ash pastes. This phenomenon was explained by application of a pozzolanic mineral additive with smaller spherical ash grains, which sealed the structure of the hardened cement paste [40]. However, there are very few studies on the effect of FBC fly-ash grain fineness and shape on porosity of cement pastes. According to Sinsiri et al. [19], the shape of fly-ash grains had a significant impact on the porosity of cement pastes with their addition. These authors showed that application of very fine fly ash from pulverized fuel fired boilers (CFA) led to a lower porosity [19,41]. Cement pastes with FBC fly ash demonstrated higher porosity and permeability [19]. Nevertheless, [10] demonstrated a beneficial effect of FBC ash application on reduced coefficients of chloride ion diffusion into the hardened mortars. The more FBC ash was used, the lower the diffusion coefficient was, even by 75%.

Due to very few reports in literature regarding the corrosive environment impact on durability of mortars with FBC fly ash, this paper includes performance of full-scope tests to determine the impact of aggressive solutions of hydrochloric acid (HCl) with a low pH reaction (pH = 0.7–2) on durability of cement–ash mortars containing FBC fly ash from lignite combustion in fluidized bed boilers and mixtures containing both FBC fly ash and siliceous fly ash (CFA) originating from combustion of hard coal in pulverized-fuel-fired boilers.

## 2. Testing Methods

### 2.1. Determination of Density and Specific Surface Area

Tests of the specific surface area of fluidized bed combustion (FBC) fly ash and siliceous fly ash (CFA) from pulverized-fuel-fired boilers, as well as cement–ash binders, were conducted in line with the PN-EN 196-6:2019-01 standard. Density of cement–ash binders was determined with a pycnometer method according to the guidelines set forth in the PN-EN 197-1:2012 standard.

### 2.2. Analysis of Particle-Size Distribution

Particle-size distribution of cement–ash binders was determined by means of a Mastersizer 3000 laser particle-size analyzer with a wet-dispersion method in a range of 0.01 to 1000 μm. Isopropyl alcohol was used as a dispersant. Measurements were performed with an accuracy of the particle analyzer of >99%.

### 2.3. Determination of Phase Composition

X-ray diffraction testing (XRD) of fluidized bed combustion fly ash (FBC) and siliceous fly ash (CFA) was performed by means of a Philips X’PertSystem X-ray diffractometer (Amsterdam, The Netherlands). The measurement was carried out in a range of 2θ angles from 5° to 60°. CuKα radiation was used. Phase identification was made based on tabular data (ICPPS-ICDD).

### 2.4. Determination of Fly-Ash Pozzolanic Activity According to the PN-EN 450-1:2012 Standard ”Fly Ash for Concrete—Part 1: Definition, Specifications and Conformity Criteria”

The pozzolanic activity of fly ashes—FBC and CFA—was determined according to the PN-EN 450-1:2012 standard by preparation of mortars containing 25% by mass of fluidized bed combustion fly ash (FBC) and siliceous fly ash (CFA) in the cement. The pozzolanic activity index was determined as the ratio of the compressive strength of cement mortars containing fly ash to the strength of the standard mortar after 28 days of curing.

### 2.5. Pozzolanic-Activity Testing According to the Frattini Method

Pozzolanicity of fly ash was also estimated in a shorter time period (up to 7 days) using the Frattini method [42]. As various types of fly ash were used (FBC and CFA), mortars were made with the following ratios: 30% by mass FBC, 45% by mass FBC, 30% by mass CFA, 45% by mass CFA, and a mixture composed of 25% by mass FBC and 20% by mass CFA. One series of samples was stored for the entire period in water with a temperature of 20 °C. The other series of samples, after three days of curing in water with a temperature of 20 °C, were taken to tanks filled with water with a temperature of 50 °C, and they were stored in those conditions for the next 4 days. Then, samples were subjected to compressive-strength testing. Results of the impact of pozzolana contained in ashes were obtained by comparing the strength of cement-based mortars with the strength of cement–ash mortars.

### 2.6. Testing Hydration Heat of Binders in Cement–Ash Pastes

Hydration heat of binders was tested by means of a C-80 isothermic microcalorimeter manufactured by the SETARAM company (Philadelphia, PA, USA) at a temperature of 21 °C. Tests were conducted for cement–ash pastes while maintaining the same sample preparation procedure. A constant water-to-binder ratio was used (w/b = 0.5). Hydration heat evolution (W) was determined, indicating occurrence of maximum values of the silica effect and the total amount of heat evolved (Q) during 72 h. The uncertainty of the test device was ±0.01%.

### 2.7. Compressive-Strength Tests of Mortars

The compressive strengths of cement–ash mortars cured in a water environment and a corrosive environment were tested in line with the guidelines of the PN-EN 196-1:2016-07 standard, on specimens with a size of 40 mm × 40 mm × 160 mm, after 7, 28, 56, 90, 180, 365, and 730 days. After 24 h of curing, samples were demolded and then stored in lab conditions at a temperature of 20 ± 2 °C for a period of 28 days until the compressive-strength testing. Next, the mortars were subjected to tests of an aggressive environment’s impact on their compressive strength. The uncertainty of the compressive-strength-test results was estimated based on the calibration uncertainty of the strength-testing device, and the uncertainty of an experimenter as a B-type uncertainty at a level of 3.3%. It should be emphasized that different kinds of uncertainties can affect the results (i.e., aleatory, epistemic, and experimental uncertainty) [43]. As strength is the main parameter in determination of the load-bearing capacity of structural elements, it should be taken into account that the uncertainty of the load-bearing capacity is related to the structural system, the randomness of material properties, and the geometric dimensions of bars, as well as the uncertainties of the model [44].

### 2.8. Tests of Mortars’ Linear and Mass Changes

The resistance of the tested cement–ash mortars to corrosion was determined based on tests of linear changes of specimens stored in hydrochloric acid (HCl) solutions of different concentrations by means of a Graf–Kaufman apparatus. Test pieces sized at 40 mm × 40 mm × 160 mm were prepared in molds divided into three compartments, in which elements for measurement of linear changes had been placed earlier. After a lapse of 24 h, test pieces were taken out of the mold and placed in vessels with distilled water for a period of 28 days. Then, samples were subjected to an aggressive environment; i.e., 1%, 3%, or 5% HCl solution, or the reference environment (distilled H_2_O). Linear changes of mortars (elongation or shortening) were determined at 4-week intervals for a period of 24 months (730 days). Changes in mass of specimens tested were also monitored. Corrosive solutions were exchanged every 4 weeks. Linear changes of samples kept in HCl solutions and distilled water were determined according to the same procedure described in the PN-B-19707:2013 standard. The measuring accuracy of the samples’ mass changes was 0.01 g, and the accuracy for the samples’ linear changes was 0.001 mm.

### 2.9. Tests of Mortars’ Porosity

Porosity testing of cement–ash mortars was performed after 365 and 730 days with the use of a PoreMaster 60 mercury porosimeter (Boynton Beach, FL USA), within a pressure range of 1 to 400 MPa. Test results were expressed in the volumetric content of pores and distribution curves of pore sizes within a range of 0.0035 μm to 1000 μm.

### 2.10. Tests of Chemical Composition

Chemical (oxide) composition of cement and fly ashes—from fluidized bed combustion (FBC) and siliceous fly ash (CFA)—was determined using an X-ray fluorescence (XRF) method on compressed samples. The WDXRF Axions mAX PANalytical (Billerica, Ma, USA) spectroscope was used.

## 3. Physical and Chemical Properties of Cement and FBC and Siliceous Ashes

**Figure 1:**
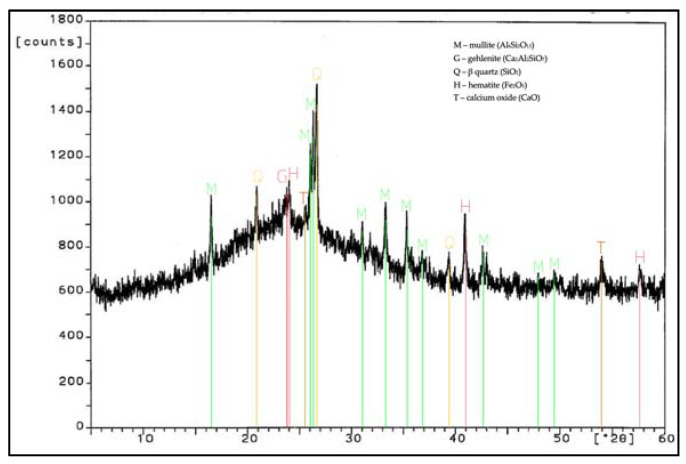
XRD pattern of siliceous fly ash (CFA) (based on results published in [45]).

**Figure 2:**
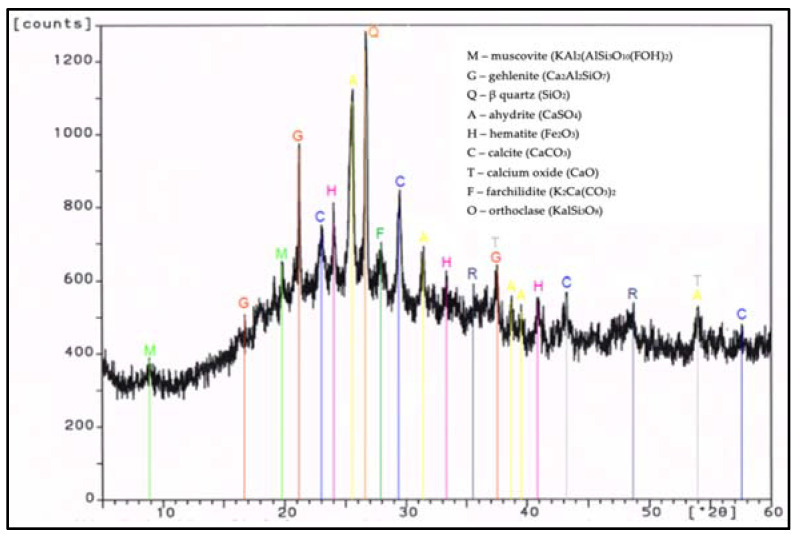
XRD pattern of fluidized bed combustion fly ash (FBC).

During work, tests of chemical composition were performed with an X-ray fluorescence (XRF) method, and on the specific surface area with a Blaine test, for CEM I 42.5 R cement, FBC fly ash, and siliceous fly ash (Table 1). Mineralogical composition of fly ash (silica and from fluidized bed combustion of lignite) is presented in diffractograms (Figure 1 and Figure 2).

Analysis of phase and chemical composition test results for FBC and siliceous fly ash (CFA) showed a difference in their compositions. The background of the diffractograms indicated a disordered amorphous structure of ashes tested (FBC and CFA). Siliceous fly ash (CFA) showed a high content of crystalline phases—such as quartz and mullite—which determined the presence of mullite, characteristic for silica fly ash. Furthermore, peaks were detected for siliceous fly ash (CFA), characteristic for hematite, calcium oxide, and anhydrite (Figure 1).

In the FBC ash, due to a lower temperature of coal combustion, no mullite was present (Figure 2). Crystalline phases like calcite, anhydrite, calcium oxide, and gehlenite dominated in the FBC fly ash. A large content of calcite in FBC ash originated from the flue gas desulfurization process, which is conducted by adding d sorbent to the boiler in a form of pulverized limestone or dolomite, whereas the presence of anhydrite in the ash was a result of a reaction between the sorbent and sulfur oxides that took place in the fluidized bed boilers.

Test results of pozzolanic activity determined after 28 days of testing (Table 2) showed that the highest pozzolanic activity was demonstrated by FBC ash; i.e., ca. 104.2%. A lower pozzolanic activity index of ca. 89% was shown by a mixture composed of fluidized fly ash (FBC) and siliceous fly ash (CFA), whereas the pozzolanic activity of siliceous fly ash (CFA) was the lowest at 78.2%. It should be emphasized that despite different results, ashes tested achieved the required pozzolanic activity index, which according to the guidelines of the PN-EN 450-1:2012 standard, after 28 days of testing, should not be lower than 75%.

**Figure 3:**
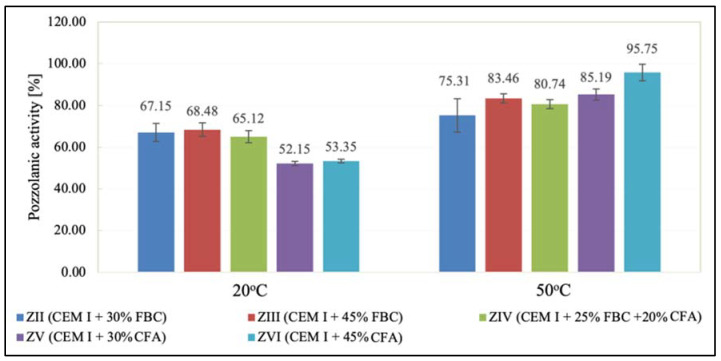
Pozzolanic activity index for fluidized-bed-combustion fly ash and siliceous fly ash determined using the Frattini method.

Pozzolanic activity tests conducted using the Frattini method (Figure 3) showed that the pozzolanic activity of FBC and CFA ashes depended on the curing temperature of specimens, and that it was higher for ashes exposed to water with a temperature of 50 °C compared to their activity determined at a temperature of 20 °C (Figure 3).

It was demonstrated that the increase of environment temperature from 20 °C to 50 °C caused a significant increase (by ca. 80%) of CFA pozzolanic activity, which was visible for ZV and ZVI mortars made of cement containing 30% and 45% by mass of those ashes, respectively.

## 4. Preparation of Mortars for Durability Tests in an Aggressive Environment

Various contents of ashes (FBC and CFA) applied (from 30 to 45% by mass) in the composition of mortars resulted in different water demands; therefore, in order to compare the properties of mortars, a constant consistency was assumed, which was tested using a Novikov slump test method (5 ± 0.5 cm).

Density and surface area of cementitious-fly-ash binders is given in Table 3. Composition of mortars tested is given in Table 4. Standard sand, which meets requirements of the PN-EN 196-1:2016-07 standard, was used as aggregate in the mortars. Fluidized bed combustion fly ash (FBC) from lignite combustion was used for testing in quantities of 30% and 45% by mass, as well as a blend composed of 25% by mass of FBC and 20% by mass CFA.

The following symbols were adopted in the study: CEM I—cement CEM I 42.5R; FBC—fly ash from combustion in fluidized bed boilers; CFA—fly ash from combustion in pulverized-fuel boilers; C—binder; Z—mortar; P—paste.

**Figure 4:**
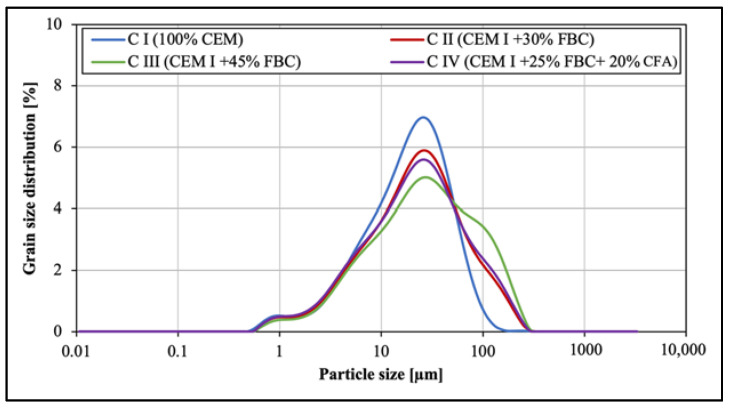
Particle-size distribution of cement–fly-ash binders.

Grain-size distribution in cement–ash binders (CI, CII, CIII, and CIV), performed by means of a laser particle-size analyzer, is presented in Figure 4 and Table 5. It was found that replacement of cement with siliceous fly ash (CFA) and fluidized bed combustion fly ash (FBC) in amounts from 30 to 45% by mass caused a significant change in the binder particle-size distribution. It led to increasing the content of fraction above 100 µm and reduction of fine fractions in the range of 10 to 50 µm. For example, the content of fine particles in the range of 10 µm to 50 µm in the CII binder with 30% by mass of FBC fly ash was higher, and amounted to 53.34% compared to the content of those particles in the CIII binder containing 45% by mass of FBC (46.6%).

In case of the CIV binder, despite it containing 45% by mass of ashes in a form of a mixture (25% by mass of FBC and 20% by mass of CFA), the content of fine particles in the range of 10 µm to 50 µm was higher (51.21%) than in the CIII binder with the same amount of FBC fly ash, but in a homogeneous form. It was characteristic that in the cases of both the CII binder and the CIV binder, the content of fine particles <50 µm and larger particles from 50 to 100 µm was comparable in the same ranges of particle sizes (Figure 4).

## 5. Interpretation of Test Results of Cement Binders and Mortar Properties with and without FBC Fly Ash and the Mixture of FBC and Siliceous Fly Ash

### 5.1. Results of Hydration Heat Testing

#### Test Results of Hydration Heat of Cementitious and Cement–Ash Binders in Pastes

**Figure 5:**
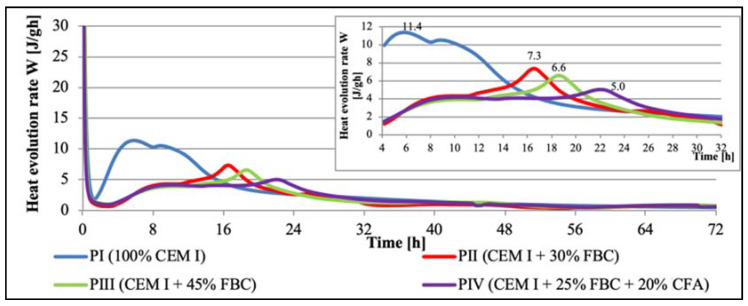
Particle-size distribution of cement–fly-ash binders.

**Figure 6:**
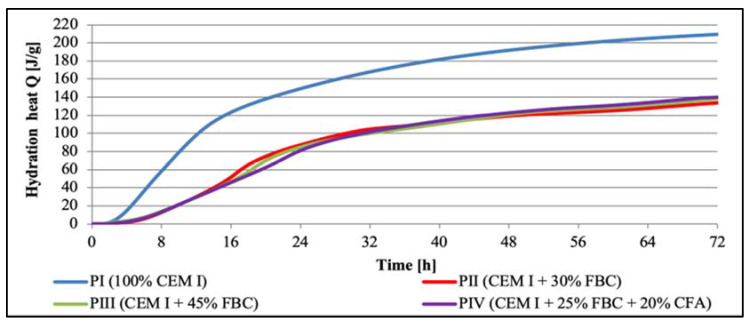
Total hydration heat evolved in cementitious and cement–ash pastes.

Test results for the hydration heat evolution rate (W) and total heat evolved (Q) for cement–ash binders in pastes are presented in Figure 5 and Figure 6. A position of maximum values of silica effects and their intensity for cements tested are summarized in Table 6 and Figure 5.

Based on the analysis of the cement hydration heat evolution rate curves (Figure 5) and the total heat evolved (Figure 6, Table 6), it may be stated that along with the increased content of fly ash in cements CII, CIII, and CIV, a significant shift of the silica effect was observed in the heat evolution rate curve toward a longer time. At the same time, reduction in intensity of the silica effect was observed, as well as reduction in the total heat evolved, compared to those parameters obtained for CEM I 42.5R Portland cement (CI), and along with the higher fly-ash content in the binder, which may be presented in the following series:


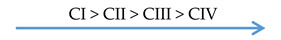


Direction of reduction: heat evolution rate and quantity, as well as maximum intensity of the silica effect in time of binder’s hydration.

The silica effect, visible in the heat evolution rate curve, was observed first for CI cement in PI paste, and it occurred only after ca. 5.8 h. Its maximum intensity amounted to 11.4 J/g·h. On the other hand, introduction of 30% by mass of FBC fly ash to Portland cement (in the CII cement) caused a shift of maximum silica effect by ca. 10 h compared to CI. That effect occurred after ca. 16.4 h of the CII cement hydration process (in PII paste) and showed the intensity was reduced to 7.3 J/g·h compared to the silica-effect intensity observed for CI cement without ash content. On the other hand, the increase of FBC fly ash amount in CIII cement to 45% by mass (PIII paste) resulted in an even higher shift of the silica effect in time, compared to the effect observed for the CI cement (in PI) and cement containing 30% by mass of FBC fly ash (in PII paste). Occurrence of the maximum silica effect for the CIII cement (in PIII paste) was found after ca. 18.8 h, and its intensity amounted to ca. 6.6 J/g·h. The largest delay of the silica-effect occurrence was observed in the case of the CIV cement containing a mixture of 25% by mass of FBC ash and 20% by mass of CFA. In the case of that binder (in PIV paste), the silica effect occurred after ca. 22 h and had the lowest intensity of ca. 5.0 J/g·h (Figure 5, Table 7).

### 5.2. Results of Compressive-Strength Tests

**Figure 7:**
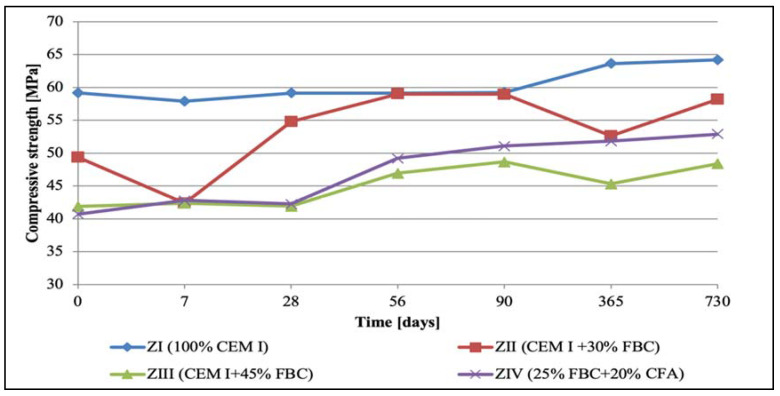
Compressive strength of mortars cured in water for 730 days.

**Figure 8:**
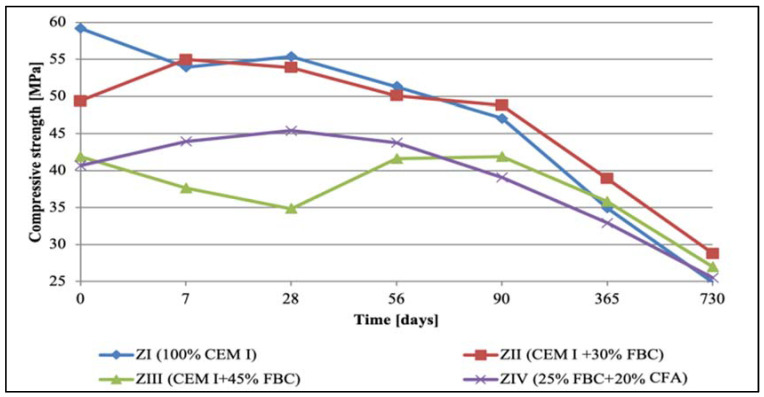
Compressive strength of mortars cured for 730 days in 1% solution of hydrochloric acid with pH = 2.

**Figure 9:**
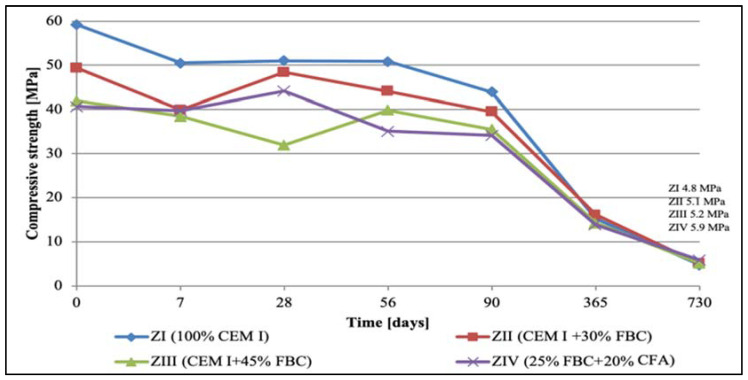
Compressive strength of mortars cured in 3% solution of hydrochloric acid with pH = 1.3 for 730 days.

**Figure 10:**
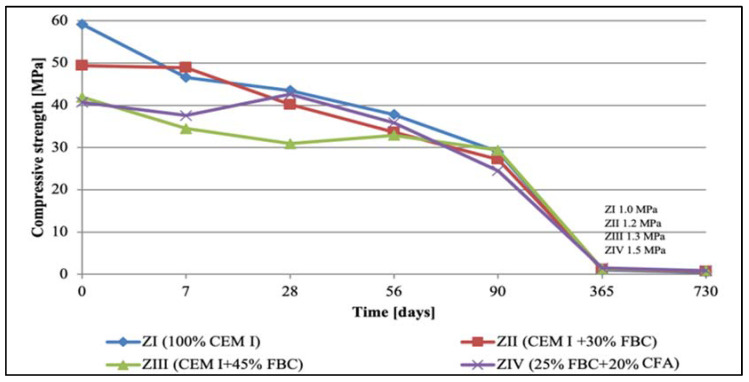
Compressive strength of mortars cured in 5% solution of hydrochloric acid with pH = 0.7 for 730 days.

The results of compressive-strength tests for cementitious and cement–ash mortars are summed up in Figure 7, Figure 8, Figure 9 and Figure 10. In the first stage of testing, the growth of the mortars’ strengths was analyzed in the period up to 28th day of their curing in a water environment (Table 8). It was found that introduction of FBC fly ash to cement, regardless of the quantity and type; i.e., used both in the homogeneous form in the smaller amount 30% by mass (in ZII mortar) and the amount increased to 45% by mass (in ZIII mortar), and also as a mixture of FBC (25% by mass) and CFA (20% by mass) (in ZIV mortar), led to reduction in the compressive strength of those mortars from 16% to 30%, compared to the strength of the reference mortar (ZI) made of CEM I 42.5 R cement (Table 8). The higher the FBC ash content in the cement, the larger the drop in strength of the mortars was. In the cases of mortars containing the same amount of fly ash; i.e., 45% by mass, but used in a different form—homogeneous in ZIII and as the blend in ZIV—the strength of ZIII mortar containing only FBC fly ash until the 28th day of curing was slightly higher than for the mortar with the ash mixture (FBC and CFA). A reverse reaction indicating a larger growth in strength, even up to 32%, was observed for the ZIV mortar containing the ash in the later time up to 730th day of testing. The above remained more in compliance with the higher pozzolanic activity of FBC fly ash (104.2%) demonstrated in tests than did the ash blend (78.2%) (Table 2). On the other hand, the lower strength of the ZIV mortar compared to the ZIII mortar up to 28th day of testing may have been affected by a higher rate of the CIII cement’s reactivity, confirmed by the higher intensity and accelerated hydration in the initial period, up to 48 h, of the hydration heat evolution rate, compared to these effects observed for CIV cement (Figure 6).

After 28 days of curing, cement (ZI) and cement–ash (ZII to ZIV) mortars were exposed to various environments, including the reference environment (distilled water) and aggressive solutions: 1%, 3%, and 5% solutions of hydrochloric acid, for a period up to 730 days.

In the water environment (H_2_O_dist._) a successive growth of the compressive strength was observed for mortars ZII, ZIII, and ZIV in the period from 28 to 730 days of testing, and the highest strength gain was shown by the ZII mortar containing 30% by mass of FBC fly ash, the strength of which was on a similar level as the strength of the ZI mortar made of CEM I 42.5 R (Figure 7). A strength lower by ca. 13% was obtained by the ZIV mortar containing a blend of 25% by mass of FBC fly ash and 20% by mass of CFA. Introduction of 45% by mass of FBC fly ash to the ZIII mortar turned out to be the least favorable, due to the smallest growth in strength compared to other mortars during the entire period of testing up to 730 days (Figure 7). In general, the compressive strength of the cement–ash mortars (ZII, ZIII, and ZIV) in the final stage of testing, on the 730th day of their storage in water, was ca. 5 to 10 MPa lower than the strength of the ZI reference mortar of CEM I 42.5 R cement.

An interesting phenomenon worth noting was that in the case of the ZII mortar with 30% by mass of FBC fly ash, a drop in the compressive strength was observed twice. The first was after only 7 days, and the other was after 365 days of storage in a water environment. Like in the case of the ZIII mortar—with 45% by mass of FBC fly ash added—the drop in the compressive strength was found after 365-day contact with distilled water. In the later period, up to 2 years of specimens conditioning in the reference environment, the compressive strength of the ZII and ZIII mortars grew again. Introduction of 45% by mass of FBC fly ash to the ZIII mortar, or 25% by mass of FBC and 20% by mass of CFA to the ZIV mortar, led to increases in the compressive strength for these mortars by approximately 2 to 3 MPa between the 365th and 730th day of testing (Figure 7).

At the same time, tests of cement–ash mortars were conducted in an aggressive environment of three solutions of hydrochloric acid with concentrations of 1, 3, and 5%, which had various pH reactions equal to: 2, 1.3, and 0.7, respectively.

Observation of the corrosive environment’s impact in case of 1% hydrochloric acid solution (pH = 2) on durability of mortars tested (Figure 8) showed that between the 90th and 365th day of testing, durability of the ZII mortar was comparable to the durability of the ZI reference mortar. The higher content of fly ash in the ZIII and ZIV mortars led to reduction of the compressive strength of those mortars compared to the strength of the reference mortar (ZI). It was noticed that in the longer period of the specimens curing (between the 90th and 730th day) in the 1% solution of hydrochloric acid, the compressive strength of the ZI reference mortar dropped at the highest rate (Figure 8). This proved a beneficial effect of application of the FBC fly ash and the blend of FBC and CFA ashes to mortars exposed to 1% HCl solution for a longer time—up to 730 days of testing.

The analysis of data presented in Figure 9 shows that up to 56 days of curing in the aggressive environment, the mortars’ strengths stayed more or less at the same level, whereas the highest drop in their strength was observed between the 90th and 365th day of testing. After 2 years of exposure of the cement–ash mortars to the aggressive environment (3% HCl), a significant reduction of their compressive strength was demonstrated. Strength values obtained after 365 days were the following: ZI, 9%; ZII, 12%; ZIII, 14%; and ZIV, 13%, compared to their original strength obtained after 28 days of curing in water (Table 8). As analyzed data show, addition of FBC fly ash in an amount of 45% by mass caused the smallest drop of the compressive strength in all mortars tested.

On the other hand, the most rapid drop in the strength was demonstrated by specimens exposed to the environment with the highest chemical aggression; i.e., the 5% solution of hydrochloric acid (HCl) with the lowest pH value, equal to 0.7. In those conditions (Figure 10), in the cases of the ZI and ZII mortars, the strength drop had occurred already by the 7th day of curing, whereas in the case when the blend of ash was used (25% FBC and 20% CFA), the strength drop was observed after 28 days; i.e., much faster than in case of the mortars stored in the environment with lower concentrations (1 and 3%) of hydrochloric acid (HCl) solution. In the case of the ZIII mortar containing 45% by mass of FBC fly ash, the compressive-strength value dropped quite suddenly, but only after 90 days of curing, as it occurred in the HCl acid solution with the lower acid concentration.

Replacement of 45% cement by mass with FBC fly ash caused a reduction in the compressive strength of ZIV mortar of 25% up to 28th day of exposure to 5% hydrochloric acid solution. After that time, a balanced level of the strength was observed up to the 90th day, when the strength went down again. After 365-day exposure to the 5% HCl solution, the strength of mortars tested was ca. 1.2 MPa. After two years of testing in the corrosive environment, all mortars (ZI, ZII, ZIII, and ZIV) achieved only a small durability, which resulted in a lack of precise determination of the compressive-strength value. Their strength values were as follows: 0.9 MPa for ZI mortar, 0.5 MPa for ZII, 0.7 MPa for ZIII, and 0.9 MPa for ZIV. Due to the measuring accuracy of the strength-testing device, it was assumed, as for lower-strength values, that mortars stored in 5% hydrochloric acid solution for 730 days showed a very low durability.

It was characteristic that in the case of the ZIII mortar containing 45% by mass of FBC ash, irrespective of the type of environment (distilled water or aggressive 1, 3, and 5% HCl solutions), a reduction in the strength was observed up to the 28th day of testing, and then after the 90th day., an increase in strength of ca. 10% in each of environments tested (Figure 7, Figure 8, Figure 9 and Figure 10) was seen. In contrast, after 90 days in the aggressive environment (1, 3, and 5% of hydrochloric acid solution), the strength of the ZIII mortar dropped again, and remained on a level comparable to other mortars. On the other hand, in the case of the ZIV mortar of CIV cement containing a similar amount of fly ash (45% by mass), but used in the form of the blend (25% FBC and 20% CFA by mass) this phenomenon was not observed. The strength of the ZIV mortar dropped faster than the strength of the ZIII mortar; i.e., after only 28 days, and it remained at the comparable level, regardless of the impact of any aggressive environment being analyzed. This proved a beneficial impact of FBC fly-ash application to the cement due to the enhanced durability of the ZIII mortar, and also in the environments with higher aggression (3 and 5% HCl; Figure 8, Figure 9 and Figure 10).

### 5.3. Test Results of Mortars’ Linear Changes

**Figure 11:**
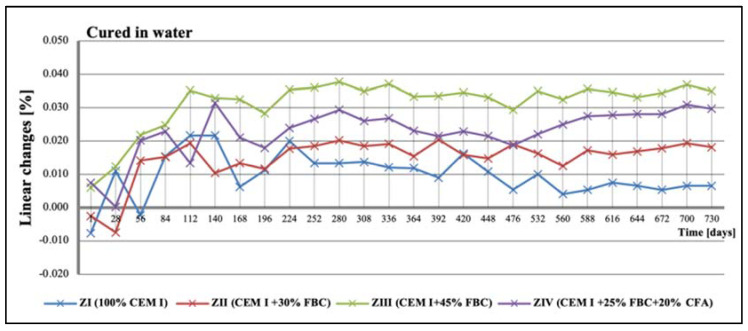
Linear changes of mortars cured in water for 730 days.

**Figure 12:**
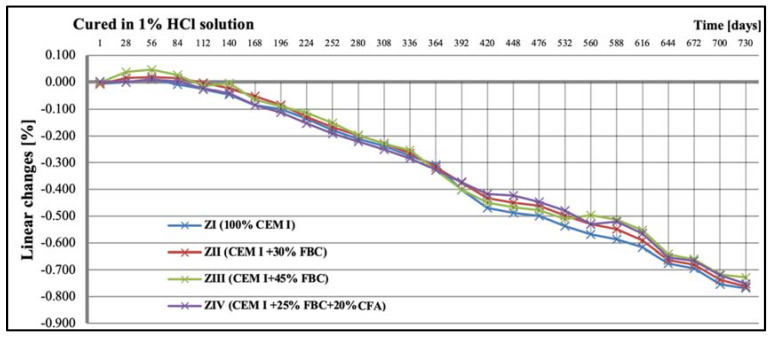
Linear changes of mortars cured in 1% HCl solution for 730 days.

**Figure 13:**
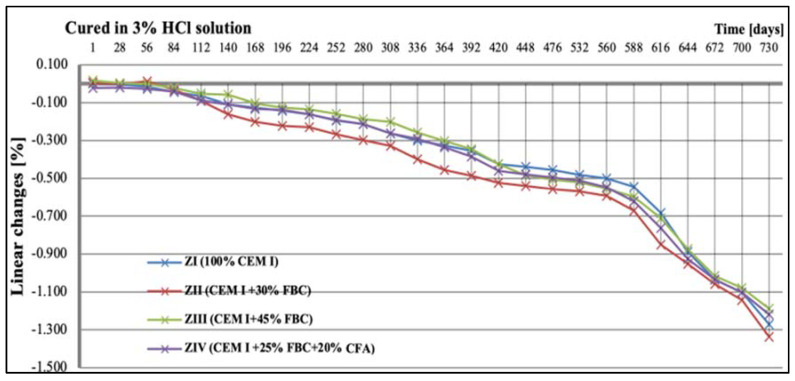
Linear changes of mortars cured in 3% HCl solution for 730 days.

**Figure 14:**
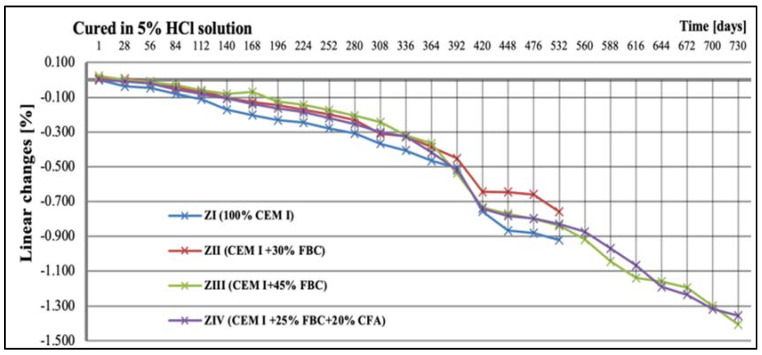
Linear changes of mortars cured in 5% HCl solution for 730 days.

**Figure 15:**
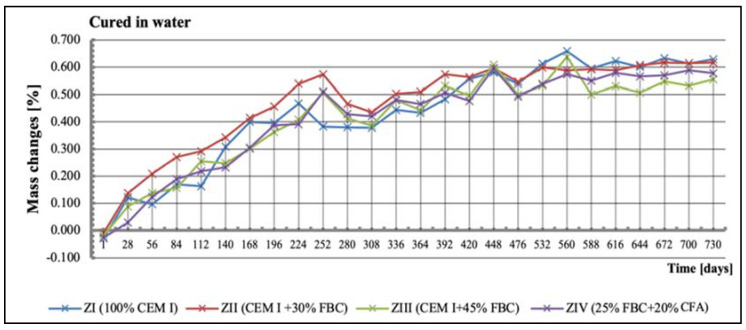
Mass changes of mortars cured in water for 730 days.

**Figure 16:**
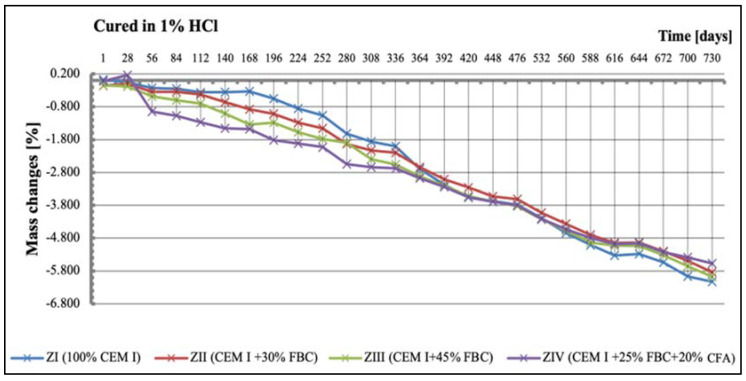
Mass changes of mortars cured in 1% HCl solution for 730 days.

**Figure 17:**
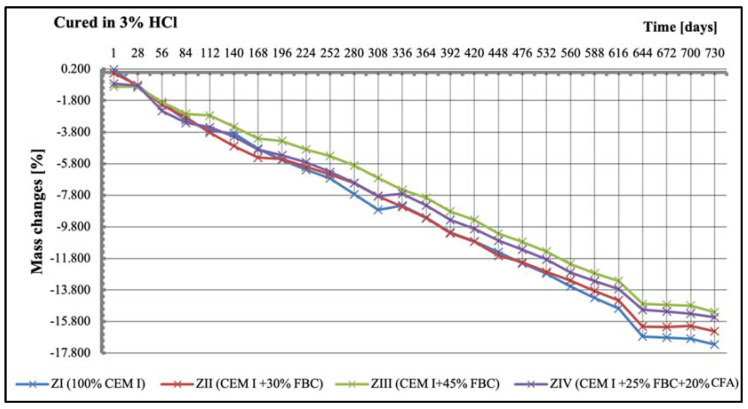
Mass changes of mortars cured in 3% HCl solution for 730 days.

**Figure 18:**
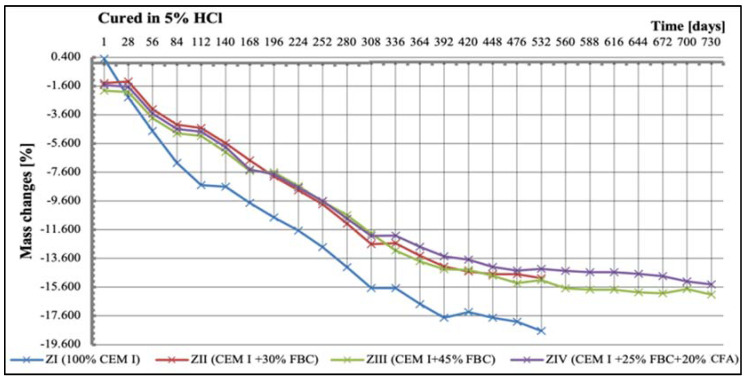
Mass changes of mortars cured in 5% HCl solution for 730 days.

Tests of linear changes of the cement–ash mortars showed mainly a reduction of the specimens’ dimensions in water (Figure 11) and the corrosive environments (Figure 12, Figure 13 and Figure 14). The above relation was also confirmed by results of mass-change tests for the mortars tested, presented in Section 5.4 (Figures 16–18). It was characteristic and interesting that the mortars tested in the reference environment (distilled water) showed mass growth (Figure 15), and also an increase in size (Figure 11), whereas in the corrosive environment, a reverse effect was observed that had an increasing tendency as the environment’s aggression grew.

Additionally, up to the 56th day of exposure to the 1% hydrochloric acid solution, an elongation of the specimens was observed (Figure 12), which, however, was not associated with the mass growth of the mortars tested (Figure 16). After that time, a reduction in the specimens’ dimensions occurred; this was observed during a two-year conditioning of the mortars, irrespective of the type and content of fly ash applied, either FBC or CFA. In general, linear changes of the mortars exposed to the 1% HCl solution were observed in the period up to 730 days, and they were comparable, regardless of their composition (Figure 12).

While in the 3% HCl solution, clear differences in the linear dimensions of the mortars were observed, depending on the quantity and type of fly ash used (Figure 13). The ZII mortar containing 30% by mass of FBC fly ash showed the greatest reduction in dimensions in the period up to two years of testing, while the ZIII mortar with 45% by mass of FBC fly ash showed the smallest linear changes out of all mortars tested. From the 588th day of testing, a rapid reduction in size for the ZI, ZII, ZIII, and ZIV mortars was noticed. At the end of the assumed time of testing; i.e., after 730 days, linear changes of the tested mortars, expressed by reduction in dimensions, amounted to over 1% (Figure 13).

It should be emphasized that the corrosive environment with the highest aggression (5% HCl solution) affected the mortar size reduction to the largest extent (Figure 14). In those conditions (5% HCl), a total degradation was observed for the reference mortar (ZI) and the mortar containing 30% by mass of FBC fly ash (ZII); therefore, tests of linear changes of those mortars were stopped after 532 days. It should be noted, however, that between the 365th and 532nd day of exposure to the 5% HCl solution, the ZII mortar samples had the smallest linear changes.

Increasing the FBC fly-ash content in the cement to 45% by mass caused the best stability of the ZIII mortar dimensions, which was confirmed by the smallest linear changes of that mortar (−0.416%) observed until 365th day of testing in those conditions (Figure 14). Mortars containing 45% by mass of fly ash (ZIII and ZIV), after a two-year period of testing, showed the lowest reduction in dimension of specimens, by ca. 1.4%, compared to their original size determined after 28 days of curing in water (Figure 14).

### 5.4. Test Results of Mortars’ Mass Changes

Results of mass-change tests for specimens exposed to various environment conditions are summarized in Figure 15, Figure 16, Figure 17 and Figure 18. It was found that as the hydrochloric acid concentration grew from 1% to 5%, simultaneous reduction of linear dimensions took place, as well as an increase in mass loss of the mortars tested (Figure 16, Figure 17 and Figure 18). In the aggressive environment; i.e., 1% HCl solution with a pH of 2 (Figure 16), the lowest mass loss was shown by the ZIV mortar. After 336 days of testing, a rapid mass loss was noticed in case of the ZI mortar, which turned out to be the greatest among the mortars tested. In the 3% HCl solution (Figure 17), the ZI mortar also showed the highest mass loss, whereas in the 5% HCl solution (Figure 18), the ZI reference mortar made of CEM I cement and the ZII mortar with 30% by mass of FBC ash underwent a complete degradation after 532 days.

The ZIV mortar containing the blend of 25% by mass of FBC ash and 20% by mass of CFA showed the lowest mass loss determined after 730-day exposure to an aggressive environment (1% and 5% HCl solution). Application of FBC fly ash in the ZIII mortar in an amount of 45% by mass, after 730 days of testing in aggressive environments of 1% and 5% (Figure 16 and Figure 18) solutions of HCl, resulted in only ca. 0.6% mass loss compared to mass loss in the ZIV mortar. In the cases of specimens cured in the 3% HCl solution, the mass losses for the ZIII and ZIV mortars were similar, and amounted to as much as 15.5% (Figure 17).

Our study also determined the cross-sectional areas of the core surfaces of mortars exposed to aggressive environments (1, 3, and 5% HCl solutions); these are illustrated in Table 9. The largest cross-sectional area of the non-corroded core of ZIII mortar obtained in HCl solution confirmed that the mortar of CEM I 42.5R with 45% by mass of FBC ash showed the highest resistance to the most aggressive environment. The measuring accuracy was ±0.05 mm. It was demonstrated that with content of FBC ash of 30% by mass in the ZII mortar, a destructive risk grew, and its resistance to acid corrosion decreased in in both the 3% and 5% HCl solutions after 365 days of testing.

This was revealed by a reduction in the surface cross-sectional area of the non-corroded core specimen for the ZII mortar, which is illustrated by the photographs and determined values of cross-sectional areas summarized in Table 9. The above was analogical to durability of these mortars expressed by their compressive strength and lower linear and mass changes (Figure 13, Figure 14, Figure 17, and Figure 18). The cross-sectional area of the core surface of the reference mortar made of pure CEM I 42.5R (without any fly ash) in the 1% HCl solution after 730 days of testing was the smallest (Table 9). The above indicated the largest corrosion progress in this mortar, also confirmed by its largest linear changes (Figure 12) and the highest mass loss (Figure 16).

It was found that the higher the content of FBC fly ash in the cement, the larger the area of non-corroded core of the mortar exposed to the aggressive environment was. This was confirmed by a surface area reduced by 0.79 cm^2^ of the core not degraded by the aggressive environment (1% HCl) of the ZIV mortar (with 25% by mass of FBC and 20% by mass of CFA) compared to the surface area of the non-corroded core of the ZIII mortar (with 45% by mass of FBC) identified after 730 days of testing (Table 9). On the other hand, in the environment with higher aggression; i.e., the 3% HCl solution, only the ZIII mortar with 45% by mass of FBC fly ash did not undergo complete degradation, and the cross-sectional area of the non-corroded core after 730-day exposure to aggressive HCl was 0.52 cm^2^. All mortars tested that were exposed to the 5% hydrochloric acid solution for 730 days showed the presence of a non-degraded area in the specimen’s cross-section, which indicated their complete destruction (Table 9).

### 5.5. Results of Mortars’ Porosity Testing

Test results of the mortars’ porosities (ZI–ZIV) obtained until the 365th day of exposure to distilled water and corrosive environments were a subject of an earlier publication [34], in which the authors analyzed in detail the resistance of mortars depending on their compositions. Due to high degradation of specimens stored in 3 and 5% solutions of hydrochloric acid, preparation of those specimens for porosity testing was impossible (the samples fell apart during preparation).

**Figure 19:**
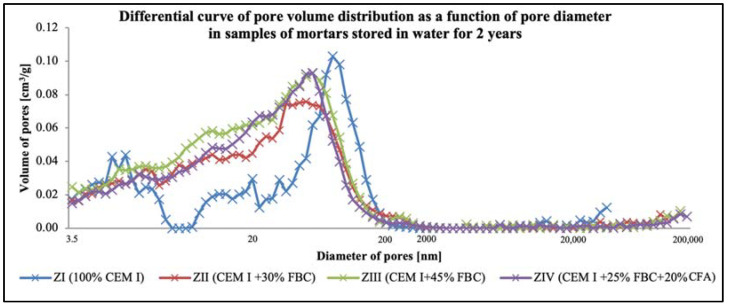
Differential curve of pore volume distribution as a function of pore diameter in samples of mortars stored in water for 730 days.

**Figure 20:**
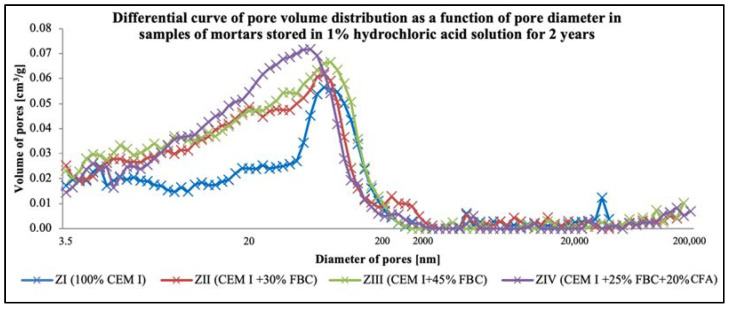
Differential curve of pore volume distribution as a function of pore diameter in samples of mortars stored in 1% hydrochloric acid solution for 730 days.

Porosity tests (Figure 19 and Figure 20, Table 10) showed that the total content of pores in the tested mortars stored in water and HCl solutions grew along with the increase of the FBC fly-ash content in the mortar. For mortars stored in the distilled water environment, after 365 days of testing, the total content of pores was: ZI mortar—11.4%; ZII—16.7%; ZIII—17.0%; ZIV—16.3%. In the case of the ZIII mortar with 45% by mass of FBC ash cured in water, no essential changes in porosity were observed in the second year; i.e., between the 365th and 730th day of testing. The ZII mortar, containing a lower amount of FBC ash in cement (30% by mass), showed a reduction in the total number of pores by ca. 1.5% in the period between the 365th and 730th day. Replacement of 20% by mass of FBC fly ash with siliceous fly ash (CFA) in the ZIV mortar also resulted in reduction of porosity by ca. 1%. It was observed in this study that the cement–ash mortars (ZII, ZIII, ZIV) showed an increased number of nanopores up to 20 nm in size, with simultaneous reduction in the number of micropores with diameters from 20–200 nm, compared to the reference mortar (ZI), which might prove the increase in tightness of those mortars (Figure 19) and thus explain their enhanced resistance to the aggressive environment.

It was observed that under the impact of the 1% HCl solution, the total number of pores in the ZI mortar made of pure CEM I Portland cement, after 730 days of testing, decreased by as much as twice (11.3%) compared to the total number of pores determined in that mortar after 365 days of testing (22.6%). In addition, in the ZII mortar with 30% by mass of FBC ash, the total content of pores decreased after two-year conditioning of that mortar specimen in the 1% HCl solution with pH = 2, and it amounted to 15.1%. The increase of fly-ash content to 45% by mass in the ZIII and ZIV mortars caused a slight reduction in the total content of pores in those mortar specimens between the 365th and 730th day of their storage in the 1% HCl solution (Table 10). After 730 days of testing, a reduced porosity was observed for the following mortars: for ZI, by ca. 50.0%; for ZII, by ca. 23.4%; for ZIII, by ca. 9.4%; and for ZIV, by ca. 13.5%. Application of fly ash to mortars exposed to the 1% HCl solution, as in case of the specimens of the ZII, ZIII, and ZIV mortars cured in water, resulted in a reduced content of pores from 20 to 200 nm in their microstructures, with simultaneous increases in the content of pores <20 nm (Figure 20).

Described changes of pore volume distribution may be explained by progressing hydration of the cementitious binder, and therefore the growth of the C-S-H phase, which fills out pores by reducing their maximum dimensions. It contributes to volume reduction of larger pores (Figure 20) and development of microporosity (increase in volume of smaller pores—Figure 20). All that takes place with a slight decrease of the total porosity (Table 10), which causes the drop of large pore content in the mortar’s porosity structure. Literature data [18] confirm that penetration of aggressive ions deep into the cement matrix is faster at the larger capillary porosity (0.09–15 μm) of the material, which was also noticed by authors of [46]. The impact of chloride ions (Cl^−^) led to decalcification of the cement matrix.

### 5.6. Results of Phase-Composition Tests of Mortars Determined after 365 and 730 Days of Curing in the 1% HCl Solution

**Figure 21:**
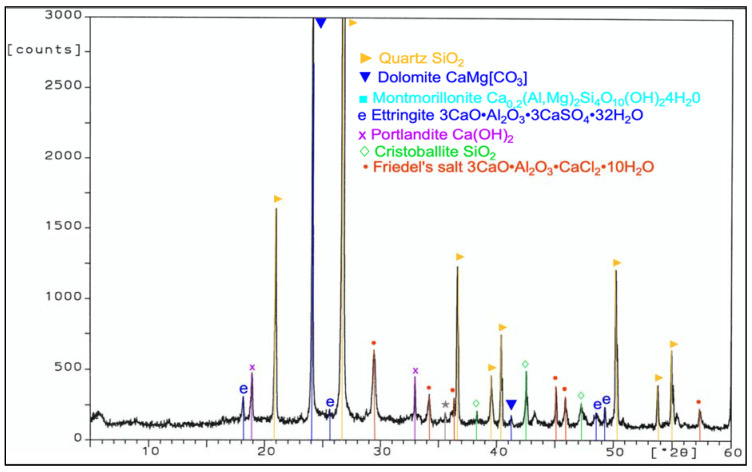
XRD pattern of the ZI mortar (100% CEM I) stored in 1% HCl for 365 days.

**Figure 22:**
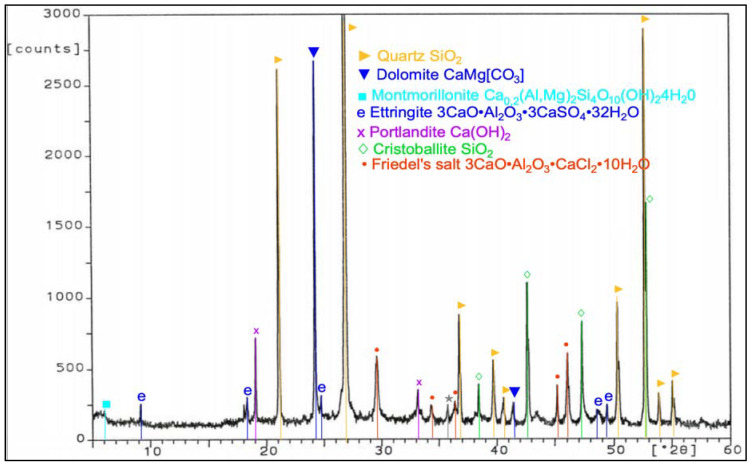
XRD pattern of the ZI mortar (100% CEM I) stored in 1% HCl for 730 days.

**Figure 23:**
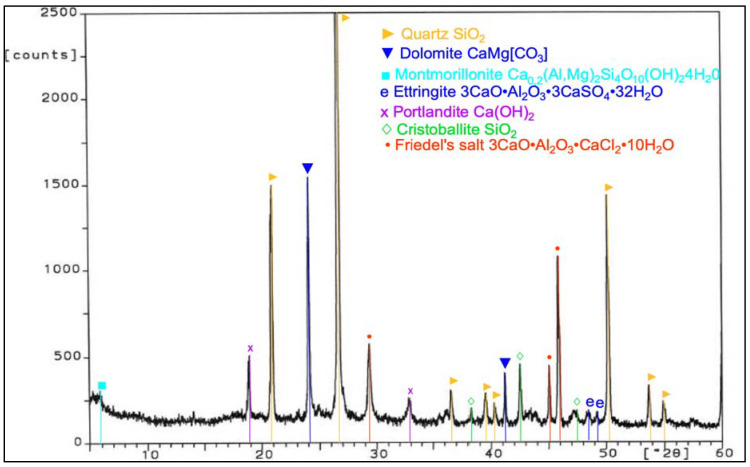
XRD pattern of the ZII mortar (CEM I + 30% FBC) stored in 1% HCl for 365 days.

**Figure 24:**
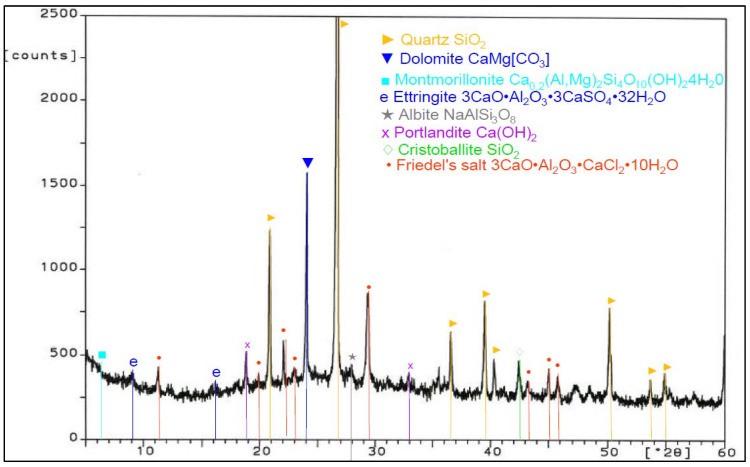
XRD pattern of the ZII mortar (CEM I + 30% FBC) stored in 1% HCl for 730 days.

**Figure 25:**
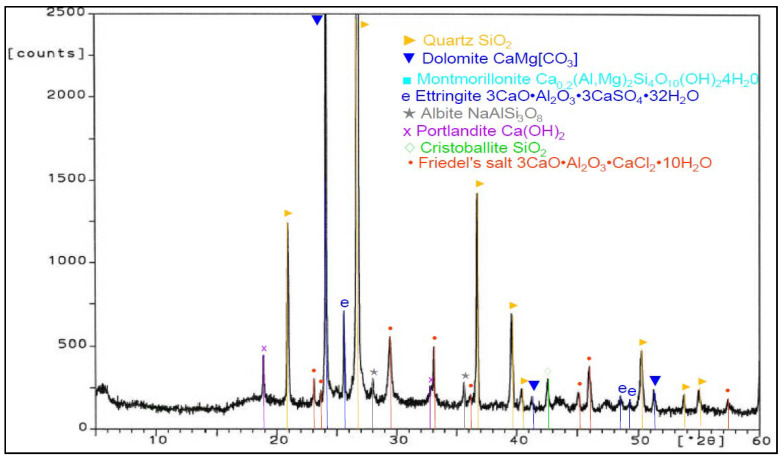
XRD pattern of the ZIII mortar (CEM I + 45% FBC) stored in 1% HCl for 365 days.

**Figure 26:**
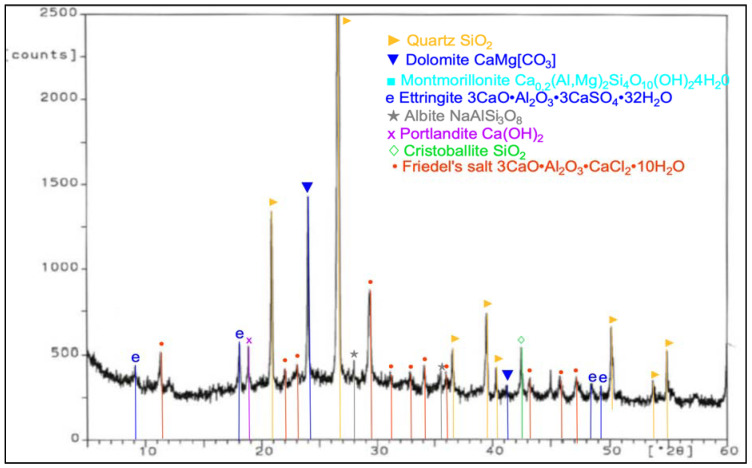
XRD pattern of the ZIII mortar (CEM I + 45% FBC) stored in 1% HCl for 730 days.

**Figure 27:**
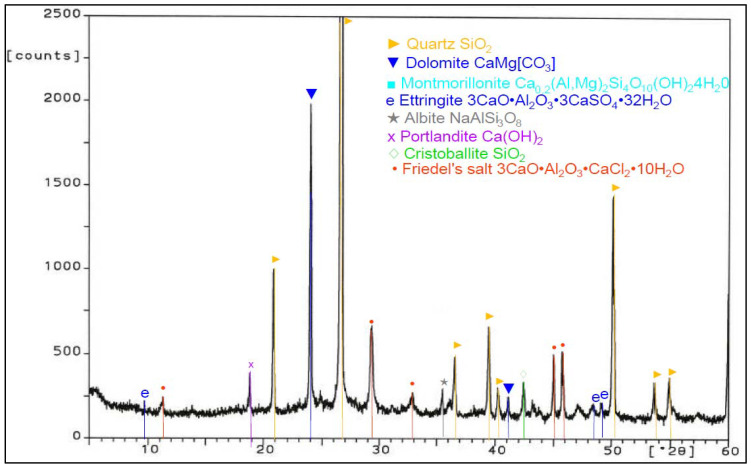
XRD pattern of the ZIV mortar (CEM I + 25% FBC + 20% CFA) stored in 1% HCl for 365 days.

**Figure 28:**
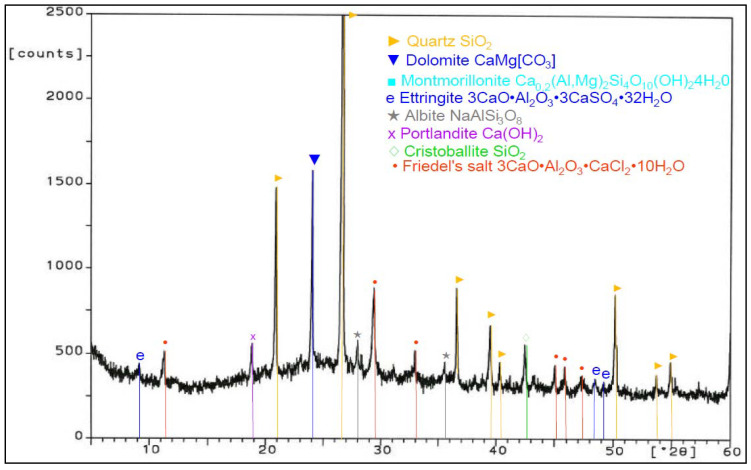
XRD pattern of the ZIV mortar (CEM I + 25% FBC + 20% CFA) stored in 1% HCl for 730 days.

Analysis of phase composition (Figure 21, Figure 22, Figure 23, Figure 24, Figure 25, Figure 26, Figure 27 and Figure 28) showed the presence of minerals characteristic of chloride corrosion; i.e., Friedel’s salt. Apart from the low pH value that affected the hardened cement–ash mortars, ion chlorides originating from hydrochloric acid had a significant impact on phase composition of the hardened mortars. Peaks typical of Friedel’s salt were observed in all samples tested. It should be noted that a peak characteristic for Friedel’s salt, assigned to 2θ angle of 11.4, after 365 days of sample storage in an aggressive environment of 1% HCl solution, was visible only in the diffractogram for the ZIV specimen (Figure 28). Only in the later period of testing (730 days) was the presence of this peak observed for all the cement–ash mortars (ZII, ZIII, ZIV).

The ZI and ZIV mortars demonstrated a presence of the peak characteristic for ettringite corresponding to 100% of intensity for the 2θ angle equal to 9.2 after 365 days of specimen storage in the corrosive environment. In the case of the cementitious mortar (ZI), this peak faded out after 730 days (Figure 22), whereas in the case of the mortar made of the blend of FBC and CFA (ZIV), that peak remained during testing (Figure 28). Application of FBC fly ash in amounts of both 30% and 45% by mass (ZII and ZIII) to mortars kept in the 1% hydrochloric acid solution affected the later crystallization of ettringite, which was proved by appearance of characteristic peaks and their increased intensity for this material after 730 days of testing (Figure 24 and Figure 26). Intensity of peaks characteristic for Friedel’s salt also increased after 730 days in all mortars tested (Figure 24, Figure 26, and Figure 28). The appearance of new peaks also was observed for Friedel’s salt after 730 days of specimen storage in an aggressive environment. In the case of the ZII and ZIII with FBC ash, reduction of the peak characteristic for portlandite was visible during testing (the peak at the 2θ angle was equal to 18.3). Such a relation was not observed for samples made of pure cement (ZI) and the blend of FBC and CFA (ZIV). It was also observed that within the 2θ angle range of 20–35 for samples made with fly ash (ZII, ZIII, and ZIV), the background of the diffractogram was raised, which proved the content of the amorphous phase in the phase composition of the mortars tested. Such a phenomenon was visible after 730 days (Figure 24, Figure 26, and Figure 28) of sample storage in a corrosive environment, and it was not observed earlier, after 365 days of testing (Figure 21, Figure 23, Figure 25, and Figure 27).

## 6. Discussion of Test Results

Tests conducted for this work confirmed that application of fly ashes from fluidized bed combustion to the cement led to an increased water demand of the binder. The same relation was observed by the authors in previous papers [29,47], and was also confirmed by research in [45,48].

The obtained results for the pozzolanic activity (Figure 3) indicated a different course of reactions occurring during the hydration of cement with FBC and CFA ashes depending on the temperature of environment. During setting of the binder at a temperature of 20 °C, a hydraulic character of ash from combustion in the fluidized bed boilers (FBC) seemed to be predominant. Increasing the environment temperature to 50 °C led to an increased rate of hydration process reactions of the cement containing siliceous ash (CFA), which, under these conditions, would indicate a predominant pozzolanic character of ashes originating from combustion in pulverized-coal-fired boilers. A similar relation was obtained in [49].

The above can be explained by a close relationship between the activity and morphology of fly-ash grains. Particles of siliceous fly ash that originated from coal combustion at temperatures of ca. 1200–1400 °C has a compact, spherical shape with a glassy structure. As a result, active oxides of silicon and aluminum, built in fly-ash particles, are hard to dissolve, and therefore they react more slowly with calcium hydroxide compared to particles of FBC fly ash [50]. At a temperature of 850 °C, clay minerals, which are the main component of coal, dehydrate and turn into a form of a dehydrated aluminosilicate substance, with a strongly disturbed internal structure and a highly developed specific surface area [10]. Therefore, reagents may diffuse very easily into FBC fly-ash grains that have a looser structure, which has a beneficial impact on the increase of the pozzolanic activity [10,50]. Furthermore, FBC fly ash particles have a different chemical and phase composition. They are mainly composed of quartz, calcium oxide, anhydrite, and gehlenite, which also affects reactivity of these ashes [24,51].

Introduction of FBC fly ash to cement in amounts of 30% and 45% by mass reduced the total heat evolved during cement hydration by ca. 15%. It was shown that the more FBC fly ash was added to the cement, the lower the total hydration heat evolved was. Application of FBC fly ash portends well for its future use as an ingredient of binders due to the effect of hydration heat reduction, in particular in conditions of increased ambient temperature. In addition, the fineness of FBC fly ash has an impact on the hydration heat of cement–ash pastes that contain these ashes. This was confirmed by results of studies performed by Zhao et al. [52]. These authors demonstrated that the increased evolution of FBC fly ash’s hydration heat depended on the fineness of the particles, which might result in destruction of the structure of the fly ashes themselves, and thus the release of soluble minerals such as Al_2_O_3_ and SiO_2_. Therefore, following the authors of [52], a reduction in the total hydration heat evolved for cement–ash pastes (Table 7, Figure 6) may be explained by the lower activity of FBC and siliceous (CFA) fly ashes compared to the higher activity of Portland cement CEM I. This also was confirmed by the results of the compressive-strength tests presented herein, which demonstrated that after 7 days of curing, the ZII mortar of cement containing 30% by mass of FBC fly ash reached 67.2% of the strength of pure CEM I 42.5 R Portland cement (in the ZI mortar).

The paper [53] also demonstrated that along with size reduction of FBC fly-ash particles contained in the cement, the increased quantity of heat evolution during its hydration was observed. These authors emphasized that application of 10% by mass of FBC fly ash with the particle size reduced to 17 µm to cement did not cause any changes in the quantity or rate of heat evolution compared to those effects observed for the pure Portland cement.

**Figure 29:**
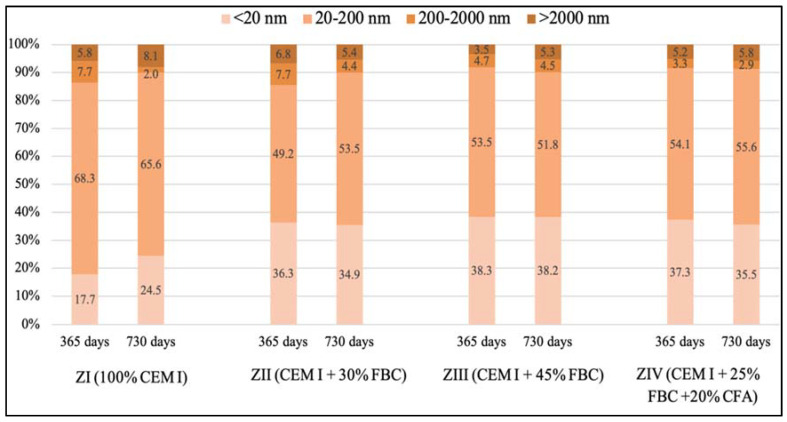
Pore size distribution for the ZI – ZIV mortars stored in water after 365 and 730 days.

The results presented herein show that the application of FBC fly ash to cement causes a reduction of hydration heat in cement–ash binders. The particle-size distribution (Figure 4 and Table 5) showed that replacement of the cement with FBC fly ash in amounts from 25 to 45% by mass caused an increased content of larger particles above 20 µm in cements CII, CIII, and CIV, in contrast to the higher content of fine particles <10 µm in the reference cement (CI) (Figure 29). Following the literature quoted, this should result in a lower total quantity of heat evolved, as well as delays in time of both setting and the silica-effect occurrence, which was in line with results of tests obtained in this work.

The compressive strength was an important issue for durability of mortars containing FBC fly ash cured in the water environment. It was shown that depending on the composition of mortars, it differed in time up to 730 days. Mortars with FBC fly ash showed a drop in their compressive strength after ca. 365 days of testing, whereas introduction of the blend of fluidized bed combustion fly ash and siliceous fly ash did not cause a decrease of the mortars’ durability in the entire period of testing, up to 730 days of conditioning in the water environment. Introduction of the blend of ashes (FBC and CFA) into the cement confirmed the possibility for wide application of such mortars, and also indicated an effective method regarding how the FBC fly ash could be improved, which was confirmed by the results presented herein and in the works of other authors [10,13].

**Figure 30:**
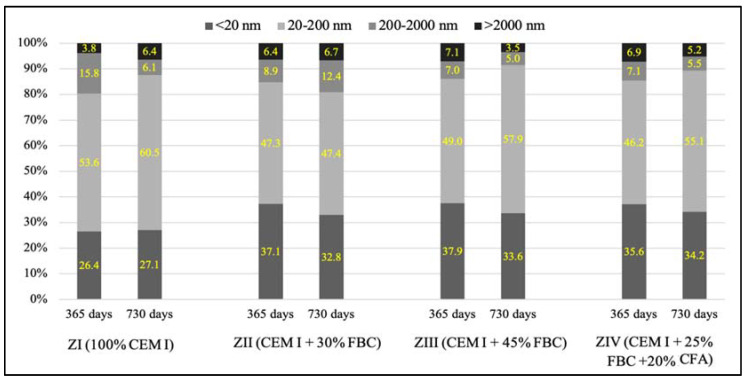
Pore size distribution for the ZI–ZIV mortars stored in the 1% HCl solution after 365 and 730 days.

It was demonstrated that the increase in durability of mortars cured in the aggressive environment of chloride ions could be achieved by increasing the content of FBC fly ash to 45%, as confirmed by test results for the ZIII mortar. The resistance of mortars with FBC fly ash in HCl solutions may be explained by the crystallization of a larger amount of Friedel’s salt, which was formed under the influence of the aggressive environment of Cl^-^ ions in the mortars [10]. The results of porosity testing presented herein were a proof of the formation of additional products in the cementitious mortars containing FBC fly ash (ZII–ZIV), the presence of which was a consequence of the reduced content of larger pores (>20nm) (Figure 30), which may be associated with a bonding reaction of chlorides with aluminum oxides [54].

Along with the progress of cement hydration, in the case of the ZI mortar specimens stored in water, there was a reduction of larger capillary pores (>200 nm) content in favor of smaller pores (20-200 nm), and the presence of mesopores (<20 nm). Similar results were presented by Chen et al. [50], as well as Tracz and Zdeb [55]. On the other hand, application of FBC ash in the cements tested did not allow us to make such a statement. For specimens of mortars (ZII–ZIV) cured in water, the total porosity was higher compared to the ZI reference mortar (Table 10). In addition, no significant changes in pore size distribution were observed in the tested samples after 365 and 730 days (Figure 29). Similar conclusions were reached by the authors of [51], who, when testing the change in pore size distribution for mortars containing up to 40% of FBC fly ash, observed that with the progress of time (up to 90 days of testing), the content of larger pores decreased in favor of smaller pores, with a slight increase in total porosity. However, the porosity test results obtained were higher than the porosity of the Portland cement during the entire period of testing [51]. A reverse relation was observed by Zhao et al. in their paper [52], in which they demonstrated that FBC fly-ash application to cement–ash pastes containing 30% FBC fly ash, resulted in the initial period in an increased number of larger pores, up to 200 nm, compared to the cementitious mortar.

**Figure 31:**
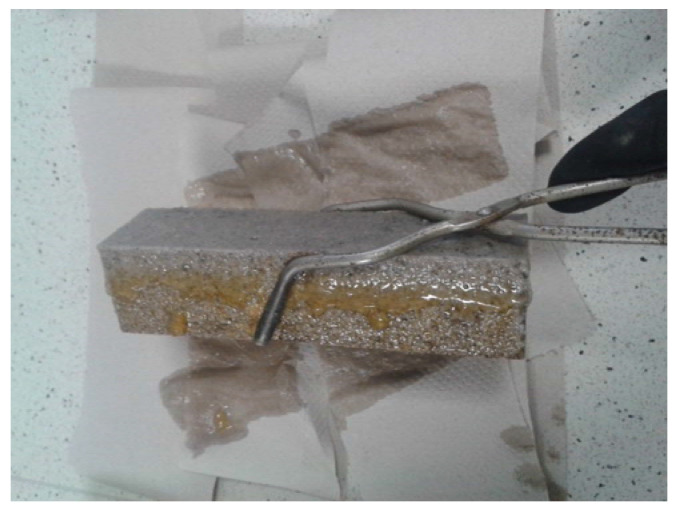
Gel substance on ZIII mortar exposed to 5% hydrochloric acid solution after 365 days.

The analyzed changes in pore volume distribution may be explained by the growth of the C-S-H phase, which fills out pores by reducing their maximum dimensions. It contributes to volume reduction of larger pores (Figure 29) and development of microporosity (increase in volume of smaller pores—Figure 30). All that takes place at a slight decrease of the total porosity (Table 10), which causes a drop in large pore content in the mortars microstructure. Literature data [18] confirm that penetration of aggressive ions deep into the cement matrix is faster at the larger capillary porosity (0.09–15 μm) of the material, which was also noticed by authors of [45]. The impact of chloride ions (Cl^−^) led to decalcification of the cement matrix (Figure 31), which had a direct effect on the reduction of the mortars’ durability.

As demonstrated in [52], the fineness of FBC fly ash has a significant impact on porosity of cement–ash pastes containing such fly ash.

A gel substance was identified on samples exposed to a corrosive environment (Figure 31), which most probably was a side-product of the C-S-H phase in a form of silicic acid gel, which is formed from the C-S-H when the pH value drops below 10 [18].

However, in the case of mortars stored in an aggressive environment (1% HCl solution), the total porosity after 730 days of testing was lower compared to their porosity demonstrated for samples stored in water (Table 10). It was shown that the use of 45% FBC and a mixture of fly ash (25% FBC + 20% CFA) in the CIII and CIV cements caused a reduction in the quantity of the largest pores (>200 nm) in the period from 365 to 730 days of exposure to the aggressive environment (Figure 31). On the other hand, the content of medium pores (20–200 nm) was increased, with simultaneous reduction in the content of the smallest pores (<20 nm).

The difference in porosity between the 365th and 730th day of testing was the smallest in the case of the ZIII mortar with the largest (45% by mass) content of FBC ash out of all tested mortars exposed to the 1% HCl solution. Replacement of FBC fly ash with siliceous fly ash (CFA) in the amount of 20% by mass did not cause a reduction in porosity of that mortar, as the results of research by Sinsiri et al. might suggest [19]. Generally, these mortars (ZII and ZIV) showed higher values of the compressive strength, lower mass changes, and increased durability in the aggressive environments of 1%, 3%, and 5% HCl solutions compared to the ZI reference mortar. Moreover, it was shown that the greater the content of fly ash in the cement, the smaller the changes in the mass of the mortar samples stored in the aggressive environment for the period of 730 days were. On the other hand, in the most aggressive environment; i.e., in the 5% HCl solution, introduction of fly ash to cement turned out to be the most advantageous due to the increased compressive strength and limited linear changes. Following Brandt et al. [10], the observed linear changes in samples containing FBC fly ash exposed to an aggressive environment can be explained by the reaction (3) of calcium hydroxide dissolution and formation of easily soluble calcium salts, the washing out of which led to weakening of the mortar microstructure:

nCa(OH)_2_ + 2 H_n_R → Ca_n_R_2_ + 2 n H_2_O (3)

**Figure 32:**
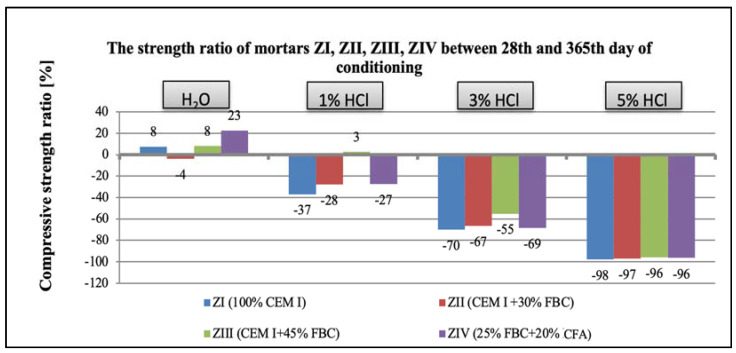
Compressive-strength ratio of mortars ZI, ZII, ZIII, and ZIV between the 28th and 365th day of conditioning in water and in 1%, 3%, and 5% solutions of hydrochloric acid.

**Figure 33:**
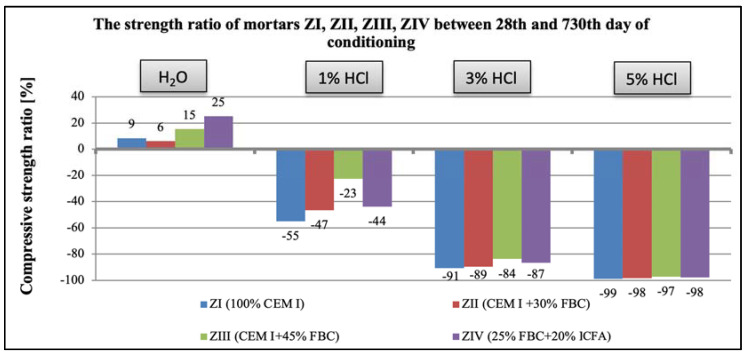
Compressive-strength ratio of mortars ZI, ZII, ZIII, and ZIV between the 28th and 730th day of conditioning in water and in 1%, 3%, and 5% solutions of hydrochloric acid.

The research results obtained in the work show that with the increase in the aggressiveness of the environment (the increase in the concentration of HCl solution from 1 to 5%), the mortar strength decreased, which was observed after both 365 and 730 days (Figure 32 and Figure 33).

Moreover, a high concentration of hydrochloric acid solution (5% HCl solution) caused the greatest linear changes in the mortars, leading in consequence to their complete destruction.

Based on the obtained results, it can be concluded that the greater the aggressiveness of the environment affecting the mortar samples with FBC fly ash, the higher the rate at which the long-term compressive strength, determined after 730 days of curing, successively decreased for all cement–ash mortars (Figure 33). However, in conditions of increased aggressiveness of the environment (5% HCl), it was possible to determine the advanced progress of destruction depending on the mortar’s composition. It was shown that mortars containing FBC fly ash had a higher compressive strength in corrosive environments, with the lowest pH = 0.7, whereas the ZI and ZII mortars with the highest amount of cement, after 532 days of storage in a 5% hydrochloric acid solution, showed complete destruction, which prevented determination of their strength parameters. Test results showed that FBC fly ash improved the corrosion resistance of mortars in the environment of the aggressive HCl solution. The study showed that the ZIII mortar with 45% by mass of FBC fly ash had the lowest drop in compressive strength. After 365 days, the strength of that mortar decreased by 55%, and after 730 days by 84%. In the 1% solution of hydrochloric acid, the increase of that strength was 3% after 365 days of conditioning, and even by 23% after 730 days.

The susceptibility of mortars or concrete made of Portland cement to corrosion resulted from two factors: porosity and the presence of calcium hydroxide—Ca(OH)_2_, which originates mainly from alite and belite. Only ca. 1–2% of calcium hydroxide originates from hydration of free lime—unbound in C_3_S, C_2_S, C_3_A, and C_4_AF. In addition, resistance of cementitious materials to corrosion in environments containing sulfate ions (SO_4_^2−^) is determined by the content of C_3_A, which can react with these ions after hydration to form salts that “burst” the cementitious material. The consequence of acid corrosion is the formation of easily soluble calcium salts, decomposition of the C-S-H phase, or decomposition of calcium carbonate in the carbonated zone [2,10,54,56].

An analysis of data in [43] indicates that as the aggressiveness of the corrosive environment increases, mortars and concretes exposed to hydrochloric acid corrosion show deterioration in strength properties and reduced durability. Capillary and gel pores in a hardened cement matrix are rich in dissolved alkali ions, calcium ions, and hydroxyl groups. Aggression caused by the hydrochloric acid is accompanied by mechanisms and reactions that lead to weakening of the cement matrix. First, there is a change in the ion concentration in pores of cementitious materials. Such an ionic imbalance causes reduction of the pH value of the cement paste in mortars and concretes, leading to three main problems: dissolution of portlandite Ca(OH)_2_ and leaching of calcium ions from hydrate and unhydrated cement clinker phases into the external solution, corrosion of reinforcement steel, and loss of strength of the cementitious material [44,57].

Studies [2,9,49,56] indicate that in the presence of chlorides from acid corrosion, deterioration in the performance parameters of mortars occurs as the aggressiveness of the environment increases.

On the other hand, research [12] indicates that the use of cement with increased SO_3_ content to mortars can delay expansion in concrete. This was also confirmed by the results of this paper, showing linear changes in the dimensions of the tested samples toward their reduction (Figure 12, Figure 13 and Figure 14). Furthermore, it has been shown that as the aggressiveness of the environment increased, durability of cement–ash mortars in an water environment decreased, whereas the use of FBC fly ash in mortars increased their durability expressed as a reduction in their expansion and an increase in their strength in the aggressive environment (Figure 7, Figure 8, Figure 9, Figure 10, Figure 11, Figure 12, Figure 13, Figure 14, Figure 15, Figure 16, Figure 17 and Figure 18).

These results were consistent with the results of [49], which showed that the presence of fly ashes from fluidized bed boilers in cement led to inhibition of calcium ion leaching from the cement matrix of mortars stored in a water environment. On the other hand, in the environment of chloride ions (Cl^−^) coming from both sea salt and an aggressive hydrochloric acid (HCl) environment, an increased amount of Ca^2+^ ions leached out, and a lack of expansive linear and mass changes in cement–ash mortars was observed.

Going further, the authors of [12] even indicated possible application of the cement with increased SO_3_ content for production of concrete elements for coastal structures. In their opinion, under these conditions, as a result of the reaction between sulfate ions and the C_3_A and C-S-H phases, ettringite is formed, which does not show expansion. These authors explained the lack of expansion by the fact that, in the presence of chlorides, calcium ions that constitute a significant part of ettringite are washed out of cement-based materials with seawater.

This also was confirmed in [17], in which the authors demonstrated that the product of the reaction of chlorides from sea salt with ettringite is the chloride equivalent of ettringite (C_3_A·3CaCl_2_·30H_2_O), which is washed out of cementitious materials containing FBC ash under the impact of seawater.

It has also been reported [58] that the introduction of FBC ash into cement, due to the high alumina content in FBC, can help mitigate corrosion of reinforcement steel by binding chloride ions from the solution in pores.

Due to few existing literature reports on the effect of FBC ash on the durability of mortars in a hydrochloric acid environment, the research undertaken here significantly complements the issues related to the corrosion of cement–ash mortars, and indicates possibilities for their application in practice. The use of FBC ash as a cement ingredient or as a mineral-additive filler in mortars or concretes has not been standardized yet. It results from the high variability of their composition during the process of their production in fluidized bed boilers, and from the increased content of sulfur trioxide (SO_3_).

Therefore, the results obtained in this study provide an opportunity for faster utilization of this troublesome waste produced in power plants or combined heat and power plants, and they confirmed the hypothesis that their application in concrete structural elements exposed to seawater or acid rain is appropriate.

## 7. Conclusions

Our research on the impact of an aggressive environment on the corrosion resistance of mortars containing siliceous fly ash and FBC fly ash was continued for a period of 2 years, and it showed that:

- The increase of the mortars’ curing temperature from 20 to 50 °C affected the increased strength of mortars, which directly resulted from the higher pozzolanic activity of ashes at the elevated temperature;
- Along with increasing content (from 30 to 45% by mass) of FBC fly ash in the cement, the water demand of the binder increased, while the addition of the mixture to cement that, apart from FBC fly ash (25% by mass), also contained siliceous fly ash (20% by mass), resulted in a reduction of the water demand of the cement–ash binder, and improved the performance parameters of the mortars;
- Application of FBC fly ash and the blend of FBC and siliceous fly ash had a beneficial effect on increasing durability and corrosion resistance of the mortars exposed to HCl solutions (1, 3, and 5%) for a period of 365 days; the above was confirmed by: higher compressive strength, lower linear changes, and reduced mass loss in these mortars compared to the cementitious mortar in the acid-corrosion environment;
- Along with a reduction of total porosity, the compressive strength of mortars tested increased, which was connected with the composition of mortars and the type of pores being formed; the higher the content of FBC fly ash, the higher the content of the mesopores (<50 nm) in the binder was, while the content of pores <200 nm was lower for mortars containing FBC fly ash and the blend of ashes, regardless of the environment in which the mortar was stored;
- The obtained test results confirmed the possibility of FBC fly-ash utilization by using it in cement–ash binders and in mixtures with other ash up to 45% by mass; production of cement–ash binders was possible in the strength classes of 32.5 or 42.5 (ZIII and ZIV);
- For mortars with FBC fly ash (ZII and ZIII) cured in water, linear changes and a decrease in strength were observed after both 28 and 365 days of testing, which proved the need to monitor the properties of cement–ash materials over a longer period of time.

## Figures and Tables

**Table 1:** Chemical composition of Portland cement (CEM I 42.5 R), fly ash from fluidized bed combustion, and fly ash from siliceous combustion.

Constituent [%]	Cement CEM I 42.5 R	Fly Ash from Fluidized Bed Combustion (FBC)	Siliceous Fly Ash (CFA)
Loss on ignition	2.57	4.30	1.81
Insoluble matter	0.46	-	-
SiO_2_	19.79	39.26	40.20
Fe_2_O_3_	2.98	3.79	2.00
Al_2_O_3_	5.76	29.37	6.00
CaO	62.28	12.04	43.20
MgO	1.71	1.79	4.70
SO_3_	2.62	3.13	0.10
K_2_O	-	1.01	-
Na_2_O	0.75	1.49	-
Specific surface area according to Blaine (cm^2^/g)	3570	8300	3800

**Table 2:** Pozzolanic activity of FBC fly ash, siliceous fly ash, and ash mixture according to PN-EN 450-1:2012.

	Fly Ash from Fluidized Bed Combustion (FBC)	Siliceous Fly Ash (CFA)	Mixture Of Fluidized (FBC) And Siliceous (CFA) Fly Ash
Pozzolanic activity (%)	104.2 ± 2.7	78.2 ± 3.3	89.0 ± 3.5

**Table 3:** Density and surface area of cementitious-fly-ash binders.

Name of Sample	CI (100% CEM I)	CII (CEM I + 30% FBC)	CIII (CEM I + 45% FBC)	CIV (CEM I + 25% FBC + 20% CFA)
Density (g/cm^3^)	3.04 ± 0.08	2.94 ± 0.06	3.03 ± 0.05	2.76 ± 0.07
Specific surface area (cm^2^/g)	3570 ± 70	3940 ± 90	3990 ± 70	3920 ± 60

**Table 4:** Composition of mortars tested.

Type of Mortar	CEM I 42.5 R (g)	FBC (g)	CFA (g)	Sand (g)	Water (g)	w/b
ZI (100% CEM I)	450	-	-		216	0.48
ZII (CEM I + 30% FBC)	346	104	-	1350	255	0.57
ZIII (CEM I + 45% FBC)	310	140	-		276	0.61
ZIV (CEM I + 25% FBC + 20% CFA)	310	77.8	62.2		247	0.55

**Table 5:** Particle-size distribution of cement–fly-ash binders.

Type of Binder	Particle-Size Distribution [%]			
	<10 µm	10–50 µm	50–100 µm	>100 µm
CI (100% CEM I)	26.53	61.96	10.58	0.93
CII (CEM I + 30% FBC)	25.12	53.34	14.00	7.54
CIII (CEM I + 45% FBC)	22.94	46.64	17.24	13.16
CIV (CEM I + 25% FBC + 20% CFA)	26.00	51.21	14.26	8.54

**Table 6:** Hydration heat of cement pastes’ tested Q [J/g].

Type of Cement Pastes	12 h	24 h	48 h	72 h
PI (100% CEM I)	97.9	149.3	191.7	209.3
PII (CEM I + 30% FBC)	29.6	81.0	122.5	139.9
PIII (CEM I + 45% FBC)	29.3	84.8	120.8	138.3
PIV (CEM I + 25% FBC + 20% CFA)	29.9	87.2	119.3	133.7

**Table 7:** The maximum reaction rate of C_3_S.

Cement–Fly-Ash Paste	PI (100% CEM I)	PII (CEM I + 30% FBC)	PIII (CEM I + 45% FBC)	PIV (CEM I + 25% FBC + 20% CFA)
Maximum reaction rate of W (J/g h)	11.4	7.3	6.6	5.0
Time (h)	5.8	16.4	18.8	22.3

**Table 8:** Compressive strength of mortars cured in water after 28 days (MPa).

Type of Mortar	ZI (100% CEM I)	ZII (CEM I + 30% FBC)	ZIII (CEM I + 45% FBC)	ZIV (CEM I + 25% FBC + 20% CFA)
Compressive strength (MPa)	59.2 ± 2.3	49.4 ± 1.8	41.9 ± 1.4	40.7 ± 1.3

**Table 9:** Changes and cross-sectional area of mortars with FBC and CFA, exposed to aggressive environments.

Time (days)	Environment	ZI (100% CEM I)	ZII (CEM I + 30% FBC)	ZIII (CEM I + 45% FBC)	ZIV (CEM I + 25% FBC + 20% CFA)
365	H_2_O	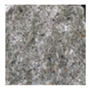	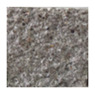	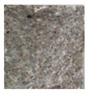	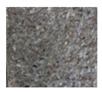
		No degradation	No degradation	No degradation	No degradation
	1% HCl	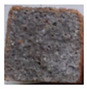	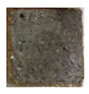	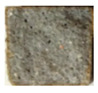	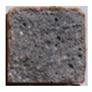
		14.5 cm^2^	14.9 cm^2^	15.6 cm^2^	Rusty rim on the surface
	3% HCl	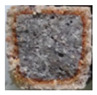	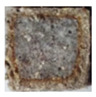	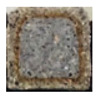	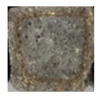
		7.60 cm^2^	8.24 cm^2^	9.36 cm^2^	8.72 cm^2^
	5% HCl	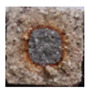	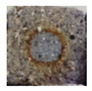	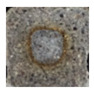	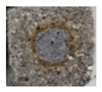
		1.86 cm^2^	1.96 cm^2^	2.04 cm^2^	2.01 cm^2^
730	H_2_O	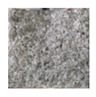	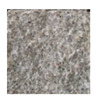	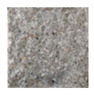	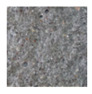
		No degradation	No degradation	No degradation	No degradation
	1% HCl	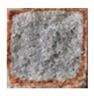	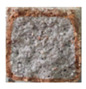	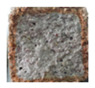	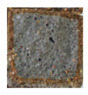
		12.34 cm^2^	13.04 cm^2^	13.85 cm^2^	13.44 cm^2^
	3% HCl	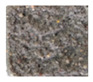	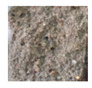	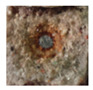	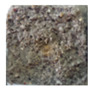
		Total degradation of section	Total degradation of section	0.52 cm^2^	Total degradation of section
	5% HCl	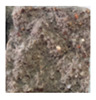	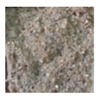	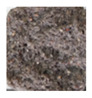	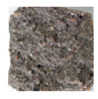
		Total degradation of section	Total degradation of section	Total degradation of section	Total degradation of section

**Table 10:** Percentage content of pores in mortars cured in water and 1% HCl solution for 365 days.

Type of Mortar	Apparent Density (g/cm^3^)	Total Porosity (%)	Percentage of Pores [%]				
			<20 nm	20–200 nm	200–2000 nm	2000–20,000 nm	<20,000 nm
H_2_O (365 days)							
ZI (100% CEM I)	2.12	11.4	17.7	68.3	7.7	0.4	5.4
ZII (CEM I + 30% FBC)	2.10	16.7	36.3	49.2	7.7	2.2	4.6
ZIII (CEM I + 45% FBC)	2.07	17.0	38.3	53.5	4.7	0.9	2.6
ZIV (CEM I + 25% FBC + 20% CFA)	2.11	16.3	37.3	54.1	3.3	0.6	4.6
H_2_O (730 days)							
ZI (100% CEM I)	2.18	13.1	24.5	65.6	2.0	0.3	7.8
ZII (CEM I + 30% FBC)	2.15	15.2	34.9	53.5	4.4	0.2	5.2
ZIII (CEM I + 45% FBC)	2.1	17.3	38.2	51.8	4.5	0.7	4.6
ZIV (CEM I + 25% FBC + 20% CFA)	2.12	15.3	35.5	55.6	2.9	0.6	5.2
1% HCl solution (365 days)							
ZI (100% CEM I)	2.15	22.6	26.4	53.6	15.8	2.5	1.3
ZII (CEM I + 30% FBC)	2.10	19.7	37.1	47.3	8.9	1.8	4.6
ZIII (CEM I + 45% FBC)	2.08	16.8	37.9	49.0	7.0	2.5	3.6
ZIV (CEM I + 25% FBC + 20% CFA)	2.09	17.1	35.6	46.2	7.1	4.2	6.7
1% HCl solution (730 days)							
ZI (100% CEM I)	2.17	11.3	27.1	60.5	6.1	1.9	4.5
ZII (CEM I + 30% FBC)	2.14	15.1	32.8	47.4	12.4	2.6	4.1
ZIII (CEM I + 45% FBC)	2.06	15.3	33.6	57.9	5.0	0.9	2.6
ZIV (CEM I + 25% FBC + 20% CFA)	2.14	14.8	34.2	55.1	5.5	0.8	4.4

## Data Availability

Data is contained within the article.
